# Development of a Sprayable Hydrogel-Based Wound Dressing: An In Vitro Model

**DOI:** 10.3390/gels10030176

**Published:** 2024-03-01

**Authors:** Mine Altunbek, Mert Gezek, Maria Eduarda Torres Gouveia, Gulden Camci-Unal

**Affiliations:** 1 Department of Chemical Engineering, University of Massachusetts Lowell, 1 University Avenue, Lowell, MA 01854, USA; mine_altunbek@uml.edu (M.A.); mert_gezek@student.uml.edu (M.G.); mariaeduarda_gouveia@student.uml.edu (M.E.T.G.); 2 Biomedical Engineering and Biotechnology Program, University of Massachusetts Lowell, 1 University Avenue, Lowell, MA 01854, USA; 3 Department of Surgery, University of Massachusetts Medical School, 55 Lake Avenue North, Worcester, MA 01605, USA

**Keywords:** sprayable hydrogel dressing, calcium peroxide, antibacterial activity, self-oxygenation

## Abstract

Hydrogel-based dressings can effectively heal wounds by providing multiple functions, such as antibacterial, anti-inflammatory, and preangiogenic bioactivities. The ability to spray the dressing is important for the rapid and effective coverage of the wound surface. In this study, we developed a sprayable hydrogel-based wound dressing using naturally derived materials: hyaluronic acid and gelatin. We introduced methacrylate groups (HAMA and GelMA) to these materials to enable controllable photocrosslinking and form a stable hydrogel on the wound surface. To achieve sprayability, we evaluated the concentration of GelMA within a range of 5–15% (*w*/*v*) and then incorporated 1% (*w*/*v*) HAMA. Additionally, we incorporated calcium peroxide into the hydrogel at concentrations ranging from 0 to 12 mg/mL to provide self-oxygenation and antibacterial properties. The results showed that the composite hydrogels were sprayable and could provide oxygen for up to two weeks. The released oxygen relieved metabolic stress in fibroblasts and reduced cell death under hypoxia in in vitro culture. Furthermore, calcium peroxide added antibacterial properties to the wound dressing. In conclusion, the developed sprayable hydrogel dressing has the potential to be advantageous for wound healing due to its practical and conformable application, as well as its self-oxygenating and antibacterial functions.

## 1. Introduction

Chronic skin wounds have a significant impact on patients’ quality of life and also pose a substantial financial burden on the global healthcare system [1,2,3]. Treating these kinds of wounds is challenging because their severity varies depending on factors such as size, location, inflammation, reactive oxygen species production, microbial invasion, and ischemic condition [4,5,6]. Hydrogel-based wound dressings offer various advantages for the treatment of wounds [7,8,9,10,11,12]. Firstly, these dressings can be easily and quickly applied to irregular or deep wounds in a controlled manner. The high water content of hydrogels helps to maintain tissue homeostasis in the wound microenvironment. Additionally, they aid in the wound healing process by providing a three-dimensional (3D) porous matrix structure that allows for gaseous exchange and facilitates the migration and proliferation of fibroblasts, neovascularization, and re-epithelialization. Furthermore, the composition of hydrogel-based wound dressings can be easily modified to protect against microbial invasion, stimulate angiogenesis, reduce inflammation, activate the local immune response, and maintain oxygen levels in the wound environment.

Naturally derived extracellular matrix-based components, such as hyaluronic acid [13,14,15,16,17], collagen [16,18,19], and gelatin [18,20], are highly appealing to provide a favorable 3D biomimetic matrix for skin regeneration. Among these, hyaluronic acid, an important component of the extracellular matrix (ECM) of skin, is known to effectively promote wound healing and accelerate skin regeneration by keeping the wound microenvironment moist and preventing inflammation [13,14,15,16]. However, its structure does not promote cell adhesion, which hinders the proper regulation of cell behavior during skin regeneration. Gelatin, a hydrolyzed form of collagen, is widely used in skin tissue engineering and wound healing [20,21]. It is commonly modified with methacrylate (GelMA) to take advantage of controllable photocrosslinking and the formation of mechanically strong, stable, and biocompatible hydrogels capable of supporting fibroblast and keratinocyte migration and proliferation for skin regeneration [22,23,24]. However, the polymeric structure of GelMA can quickly degrade before healing [20,25]. Therefore, the durability of GelMA hydrogels can be robustly adjusted using different strategies, including increasing material concentration or crosslinking density and blending with other ECM components [24,25,26]. For instance, the addition of methacrylate-modified hyaluronic acid (HAMA) significantly increased the mechanical strength of GelMA hydrogels [25]. Additionally, the composite hydrogel of GelMA and HAMA showed promise for cell proliferation and vascularization, which are essential for wound healing and skin regeneration [26]. Moreover, hydrogels with higher concentrations of GelMA demonstrated resistance to collagenase degradation and provided a more suitable environment for keratinocyte adhesion, monolayer formation, and stratified epidermal layer formation [24]. These observations highlight the critical role of GelMA and HAMA composition in the functionality of the hydrogel for wound healing and skin regeneration.

Wound healing is a well-synchronized dynamic process that requires sufficient energy for various cellular applications such as cell migration, proliferation, ECM production, re-epithelization, and angiogenesis [27]. Oxygen delivery becomes crucial for meeting the energy demands of the cellular processes, as oxygen supply is limited to a depth of 3–4 mm below the tissue, and a hypoxic condition can delay wound healing [28]. To enhance wound healing, researchers are actively studying the integration of controlled and sustained oxygen release systems into treatment approaches [28,29,30,31,32,33,34,35]. One promising approach is the use of catalase-based oxygen-generating delivery systems, which take advantage of their abundant release during the wound healing process [36,37]. Catalase primarily functions to catalyze the breakdown of hydrogen peroxide (H_2_O_2_) into oxygen and water. Building on this principle, researchers have extensively explored the use of oxygen-generating materials and controlled delivery systems for wound healing and tissue engineering applications [30,34,38]. Solid peroxides, particularly calcium peroxide (CaO_2_), stand out as the most extensively investigated materials for oxygen release due to their advantageous features, including ease of accessibility, processability, and the ability to regulate the oxygen-releasing profile by controlling its decomposition into calcium and hydrogen peroxide (Ca(OH)_2_) in aqueous media [34,36,39]. The released H_2_O_2_ can also exhibit antibacterial activity, making CaO_2_ useful in overcoming another major challenge for wound healing therapies. CaO_2_ has shown potential in wound healing applications, and its beneficial effects on cell proliferation, wound closure, angiogenesis, and antibacterial and anti-inflammatory properties have been demonstrated [28,30]. However, the excessive release of H_2_O_2_ may induce oxidative stress and damage cells [40]. Therefore, convenient approaches need to be developed for the controlled use of CaO_2_ in clinical wound healing applications.

In this study, we developed a sprayable hydrogel-based wound dressing with oxygen-releasing and antibacterial properties for potential use in wound healing. We selected composite hydrogels made from a methacrylated form of gelatin (GelMA) and hyaluronic acid (HAMA) for rapid and controllable photocrosslinking and efficiently covering the wound once applied. GelMA was selected to facilitate cell migration, adhesion, and spreading, while HAMA was used to provide elasticity and structural strength to the dressing. We also incorporated bioactive CaO_2_ into the dressing to achieve multiple functions, including self-oxygenation and antibacterial activity. Additionally, the released calcium can further aid in promoting the wound healing process by regulating the behavior of keratinocytes and fibroblasts, as well as by modulating angiogenesis. We successfully developed a composite hydrogel composition that can be conveniently applied through spraying and crosslinked through photopolymerization. This sprayable hydrogel is portable and can be easily applied to irregularly shaped and deep wounds in situ. It releases oxygen to the wound area, promotes cell proliferation, decreases the cell death rate under hypoxia, and exhibits antibacterial properties. We believe that the mixture of GelMA and HAMA-based hydrogels with oxygen-releasing and antibacterial properties have considerable potential as skin wound dressings. Furthermore, it is anticipated that the composition of hydrogel-based wound dressing can potentially exhibit anti-inflammatory and vascularization properties in in vivo applications.

## 2. Results and Discussion

### 2.1. Development of the Sprayable Composite Hydrogel

**Figure 1:**
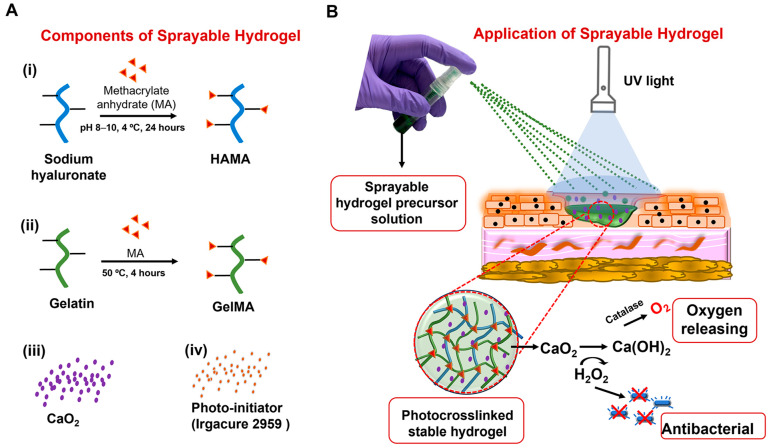
Schematic representation of the development and application of a sprayable hydrogel-based wound dressing: (**A**) The components of the sprayable hydrogels: (i) Sodium hyaluronate and (ii) gelatin modified with methacrylate anhydrate were used in the hydrogel precursor solution for easy application through photocrosslinking in the wound area. (iii) Calcium peroxide (CaO_2_) was added to the precursor solution to provide self-oxygenation and antibacterial properties, while (iv) Irgacure 2959 was included to initiate photocrosslinking when exposed to UV light. (**B**) The application and functionality of the hydrogel-based wound dressing are presented. The precursor solution was sprayed onto the wound surface. UV light was then used to crosslink and form a stable hydrogel in the wound area. The decomposition of CaO_2_ supplies oxygen and provides antibacterial activity.

The composition of biomaterials in our hydrogel wound dressings exhibited the following properties: sprayability, stable hydrogel formation, self-oxygenation, antibacterial properties, and cytocompatibility. Naturally derived polymers, including hyaluronic acid and gelatin, were selected for the formulation (Figure 1A). These polymers have been shown to effectively maintain homeostasis in the wound microenvironment and promote regeneration by enhancing cell migration and proliferation [25]. To enable instantaneous in situ crosslinking upon exposure to UV light and form a stable hydrogel at the target site, these biomaterials were modified with methacrylate groups (HAMA and GelMA) (Figure S1). Varying amounts of calcium peroxide (CaO_2_) were introduced into the hydrogel precursor solution to provide self-oxygenation and antibacterial properties for the hydrogel wound dressing (Figure 1B).

The multifunctional hydrogel was designed to be administered through spraying due to several advantages, including portability, ease of application to wounds, and the ability to modify the composition to enhance the healing of deep wounds. The composition of the selected biomaterials in the precursor solution was evaluated first to ensure compatibility with spraying. HAMA was used at a concentration of 1% (*w*/*v*), which is within the range known to be favorable for skin hydration [13,41,42]. On the other hand, determining the optimal GelMA concentration in the precursor solution was particularly critical for achieving the sprayability of the composite hydrogel. This is because the viscosity of the GelMA precursor solution is highly dependent on material concentration, which affects the flow rate at temperatures ranging from 37 °C to 45 °C [43,44]. A high viscosity or rapid gel transition can cause a decrease in the flow rate or blockage of the nozzle, resulting in poor spray coverage or application failure. The choice of a compatible nozzle can also impact spray performance, as the ejection characteristics of the nozzles vary based on the size of the tube.

**Figure 2:**
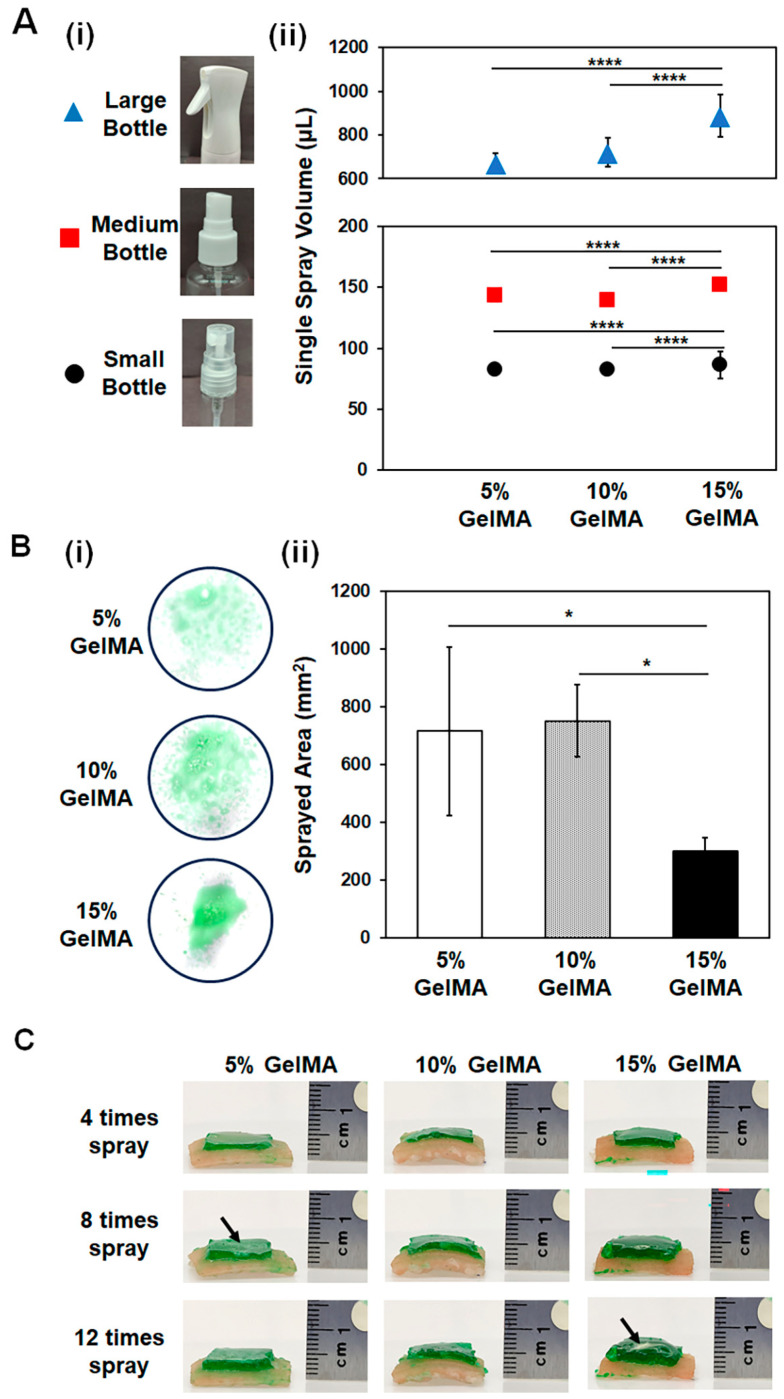
Characterization of spray for varying concentrations of GelMA hydrogel precursor solutions: (**A**) (i) Effect of various spray bottles including small, medium, and larger sizes on single spray volume of GelMA precursor solutions with concentrations of 5%, 10%, and 15% (*w*/*v*) and (ii) quantitative analysis results. (**B**) (i) Images of the single spraying area and (ii) quantitative analysis of the spraying area. (**C**) Spraying of 5%, 10%, and 15% (*w*/*v*) GelMA precursors on pig skin layer by layer. Side views of the hydrogels with varying numbers of sprays were provided to demonstrate the resulting stable hydrogels on the pig skin. Significant change between the groups was analyzed using one-way ANOVA which is presented as *p* < 0.05 (*), and *p* < 0.0001 (****).

We first assessed the efficiency of dispensing various GelMA precursor solutions in different concentrations using spray systems with different bottle sizes: large, medium, and small (Figure 2A(i)). Based on previously published reports, we conducted an investigation on GelMA concentration in the precursor solution at 5%, 10%, and 15% (*w*/*v*). Our goal was to determine the most effective concentration for achieving sprayability and the formation of a functional hydrogel that exhibits suitable swelling, and mechanical properties for skin regeneration [9,22,45]. The precursor solutions were adjusted to a temperature of 37 °C and sprayed five times into an empty tube. We used micropipettes to measure the volume, and the average of the five sprays was calculated to determine the single spray volume for each GelMA condition (Figure 2A(ii)). All hydrogel precursors were successfully sprayed without nozzle clogging. As expected, the volume of the precursor solution for a single spray increased proportionally with the size of the bottle. Regardless of the bottle size, the single spray volumes of 5% and 10% (*w*/*v*) GelMA were similar, while a significant increase was observed in the volume of the 15% (*w*/*v*) GelMA precursor solution sprayed. This increase is anticipated due to the higher viscosity of the 15% GelMA precursor at the same temperature.

The images in Figure 2B(i) suggest that the viscosity of GelMA is crucial for the stream quality and droplet size formation. GelMA precursors with concentrations of 5% and 10% (*w*/*v*) resulted in smaller droplets and a larger surface area covered per spray. Conversely, as the viscosity of GelMA increased with higher material concentration (15%, *w*/*v*), a significant change occurred, resulting in larger droplet sizes per spray and a smaller surface area coverage. The quantitative analysis shown in Figure 2B(ii), confirmed that the sprayed area decreased by approximately 50% when a 15% (*w*/*v*) GelMA precursor solution was used. There was no significant difference in the sprayed area between 5% and 10% (*w*/*v*) GelMA precursors when using the small spray bottle, but the 10% (*w*/*v*) GelMA was found to be more evenly distributed in the targeted area. This concentration of hydrogel was also found to be effective in the base formulation of the sprayable GelMA hydrogel in a previously published report [9].

Pig skin was purchased to evaluate the spray characteristics of various GelMA precursor solutions on an ex vivo skin. We applied the GelMA precursor solutions to the pig skin as follows: A manual pressure was applied to the nozzle, and the GelMA precursor solution streamed toward the pig skin. Once the target surface was covered after spraying, UV light was used for 20 s to chemically crosslink the methacrylated precursors and form a stable hydrogel (Figure 2C). We used a small size spray bottle and applied four sprays (approximately 300–375 µL) to a 1 cm^2^ area of pig skin. These spraying and crosslinking processes were repeated two more times to demonstrate the layer-by-layer application of the multifunctional hydrogel. The concentration-dependent viscosity of the hydrogel precursor was found particularly important for the application on the skin surface, as the less viscous hydrogel precursor led to quick spreading and leakage before crosslinking, while it was difficult to evenly spread the high-viscosity precursor solutions on the wound area (Figure S2). Although we observed the formation of a stable hydrogel structure with increased thickness for each layer, regardless of the concentration, when using 5% or 15% (*w*/*v*) concentrations of GelMA precursors, the distribution on the wound surface was uneven and resulted in the formation of an irregular hydrogel structure (indicated by black arrows) (Figure 2C). It is important to note that the temperature of the hydrogel precursor can be adjusted based on the GelMA precursor solution. A heat jacket can be used to maintain the precursor solution at the desired temperature for a uniform stream. In this study, we used a 37 °C incubator to keep all hydrogel precursors in solution form. Additionally, this temperature is physiologically relevant and does not cause any harmful effects on the cells in the wound area. Out of the different concentrations, we selected 10% (*w*/*v*) GelMA and mixed it with a 1% (*w*/*v*) HAMA solution for further analysis of the sprayable hydrogel. The addition of 1% (*w*/*v*) HAMA did not affect the spraying properties of the 10% (*w*/*v*) GelMA (Figure S3).

### 2.2. Characterization of the Sprayable Composite Hydrogels

**Figure 3:**
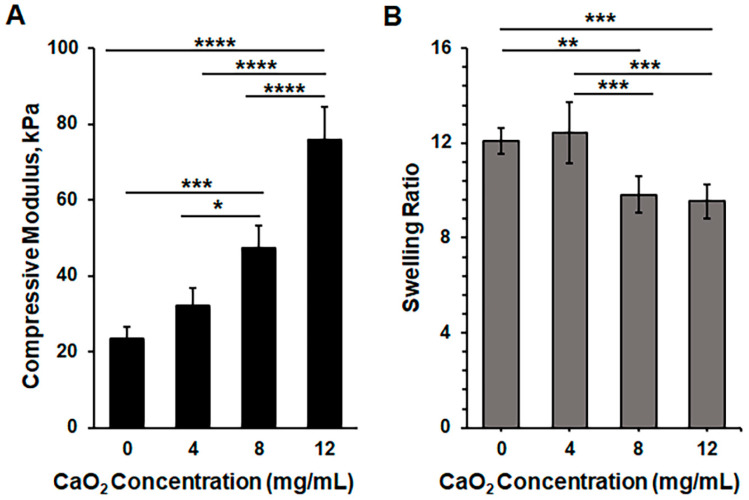
Characterization of 10% GelMA + 1% HAMA (*w*/*v*) composite hydrogels in the presence of varying amounts of CaO_2_: (**A**) compressive modulus and (**B**) swelling analyses of the hydrogels. Significant change between the groups was analyzed using one-way ANOVA which is presented as *p* < 0.05 (*), *p* < 0.01 (**), *p* < 0.001 (***), and *p* < 0.0001 (****).

To provide self-oxygenating and antibacterial properties to the sprayable hydrogel wound dressing, we added various concentrations of CaO_2_ to the precursor solutions. The presence of CaO_2_ in the precursor solution caused a change in the transparency of the hydrogel precursor solution. This change in turbidity hindered the penetration of UV light through the hydrogel precursor. As a result, we increased the exposure time to UV light in order to fully crosslink the GelMA and HAMA in the presence of CaO_2_. We characterized the mechanical and swelling properties to evaluate the effects of composition-based changes on the physical properties of the composite hydrogels (Figure 3). The compressive moduli were found to be 23 ± 3 kPa, 32 ± 5 kPa, 47 ± 6 kPa, and 76 ± 9 kPa for the pristine hydrogel (containing 0 mg/mL CaO_2_) and composite hydrogels containing 4, 8, and 12 mg/mL concentrations of CaO_2_, respectively (Figure 3A). As can be seen, there were no significant changes between the compressive moduli of the pristine hydrogel and the hydrogel containing 4 mg/mL CaO_2_, while 8 and 12 mg/mL concentrations of CaO_2_ significantly increased the mechanical strength of the hydrogels. The swelling ratios were 12.1 ± 0.53, 12.4 ± 1.28, 9.8 ± 0.77, and 9.5 ± 0.71 for the pristine composite hydrogel (0 mg/mL) and hydrogels containing 4, 8, and 12 mg/mL concentrations of CaO_2_, respectively. Conversely, the swelling ratio decreased as the CaO_2_ concentration was increased to 8 and 12 mg/mL concentrations (Figure 3B). Regardless of CaO_2_ concentration, the swelling ratio exhibited typical swelling behavior of hydrogel-based wound dressings from the literature, which can help to maintain homeostasis in the wound area and absorb the wound exudates [46,47,48]. As can be observed, increased CaO_2_ concentration induced distinct properties for the 10% GelMA + 1% HAMA composite hydrogels, similar to the findings in the literature [36].

### 2.3. Development of an In Vitro Culture Model to Analyze the Self-Oxygenating Properties and Cytocompatibility of Sprayable Composite Hydrogels

**Figure 4:**
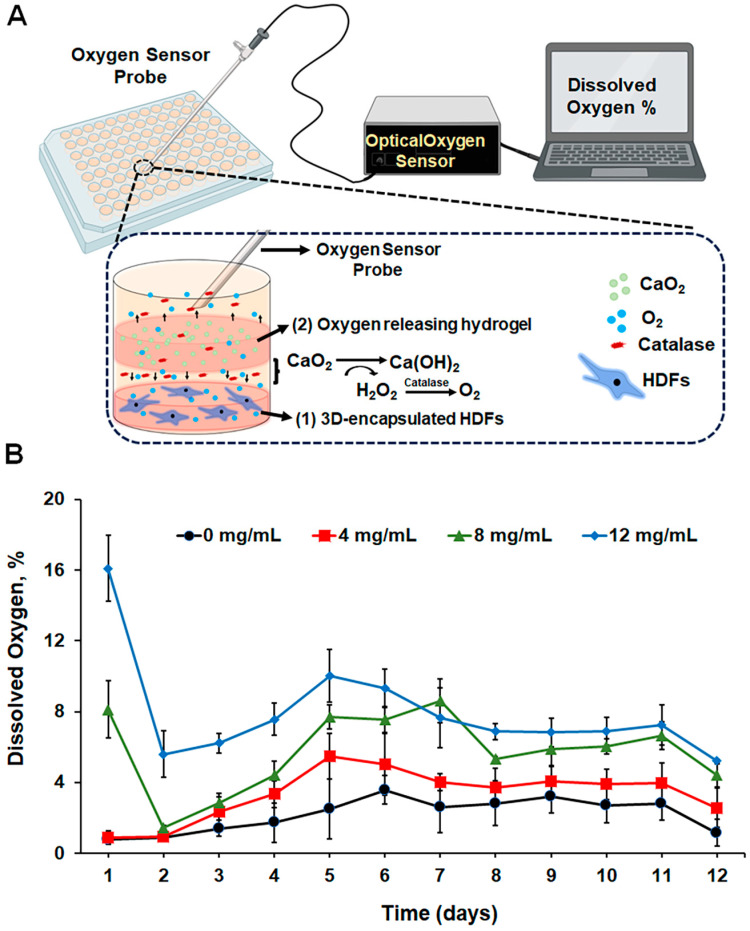
Development of an in vitro culture model to analyze the self-oxygenating properties and cytocompatibility of the sprayable composite hydrogels: (**A**) Schematic representation of an in vitro model for testing the oxygen-releasing kinetics of a sprayable multifunctional hydrogel. (1) HDFs encapsulated in a 5% GelMA (*w*/*v*) solution were placed at the bottom of a 96-well plate, and (2) composite hydrogels containing 10% GelMA + 1% HAMA (*w*/*v*), with varying concentrations of CaO_2_, were placed on top of the HDF-encapsulated hydrogel. The cell culture media were supplemented with 1 mg/mL of catalase to facilitate the dismutation of released H_2_O_2_ into oxygen. Oxygen release from the hydrogel into the cell culture media is demonstrated with up and down black arrows. Created with BioRender.com. (**B**) The in vitro oxygen release kinetics of the hydrogels were measured over 12 days using the NeoFox fluorophore oxygen probe. Daily measurements were taken to determine the percentage of oxygen dissolved in the media.

Fibroblast cells, which are located in the dermal layer of skin tissue, play a crucial role in synthesizing the ECM and regenerating skin during wound healing. The migration, proliferation, and ECM synthesis of fibroblasts are heavily regulated by the interaction between cells and the ECM in a 3D matrix [49]. As a result, hydrogel-based 3D culture models are proven useful in assessing fibroblast functions [50,51,52]. In this study, we developed an in vitro 3D hydrogel-based culture model to examine the self-oxygenating and cytocompatibility properties of the composite hydrogel. The purpose of this model is to mimic the behavior of fibroblasts in the wound area and their interaction with the hydrogel wound dressing. Figure 4A provides a schematic representation of the model: Two separate hydrogels were prepared for the development of the mimetic model. First, human dermal fibroblasts (HDFs) were encapsulated in a 5% (*w*/*v*) GelMA hydrogel at the bottom of a 96-well plate. Second, 10% (*w*/*v*) GelMA + 1% (*w*/*v*) HAMA with varying concentrations of CaO_2_ were prepared and placed on top of the 5% (*w*/*v*) GelMA hydrogels, where HDFs were encapsulated in a 96-well plate. To neutralize H_2_O_2_ into H_2_O and O_2_ upon the decomposition of CaO_2_, catalase was added to the cell culture media. The released O_2_ is expected to relieve the stress on HDFs under hypoxia, thereby maintaining cell viability and functionality. In this study, we used concentrations of 4, 8, and 12 mg/mL of CaO_2_ in the hydrogels. After preparing the model in the 96-well plate as described, the plate was placed in a hypoxia chamber, and daily oxygen measurements of the media were taken to evaluate the oxygen-releasing kinetics from the hydrogels over a two-week period (Figure 4B). For the pristine hydrogel (0 mg/mL CaO_2_), the oxygen level obtained during the daily measurements was between 1% and 2.5%. The oxygen level slightly increased in the presence of 4 mg/mL CaO_2_, but this increase was not significant compared to the control group. A burst of O_2_ release was observed on day 1 in the medium containing hydrogels with 8 and 12 mg/mL of CaO_2_, and the dissolved oxygen levels on day 1 were measured at around 8% and 16%, respectively. A sudden decrease was observed on day 2, but the level of dissolved oxygen was still higher with the use of 12 mg/mL CaO_2_-containing hydrogels. A gradual increment was observed until day 5, and the dissolved oxygen level was measured at around 6–7.5% for 8 mg/mL CaO_2_-containing hydrogels and 7.5–10% for 12 mg/mL CaO_2_-containing hydrogels. On day 8, oxygen levels were measured as 2.8%, 3.7%, 5.3%, and 6.9% in the wells where the pristine hydrogel and hydrogels containing 4, 8, and 12 mg/mL of CaO_2_ were found, respectively, and no significant differences were observed until day 11. A sudden decrease was observed on day 12, and the values were measured as 1.2%, 2.6%, 4.4%, and 5.2%, respectively. As seen, depending on the concentration of CaO_2_ in the hydrogels, the oxygen level in the cell culture media proportionally changed.

**Figure 5:**
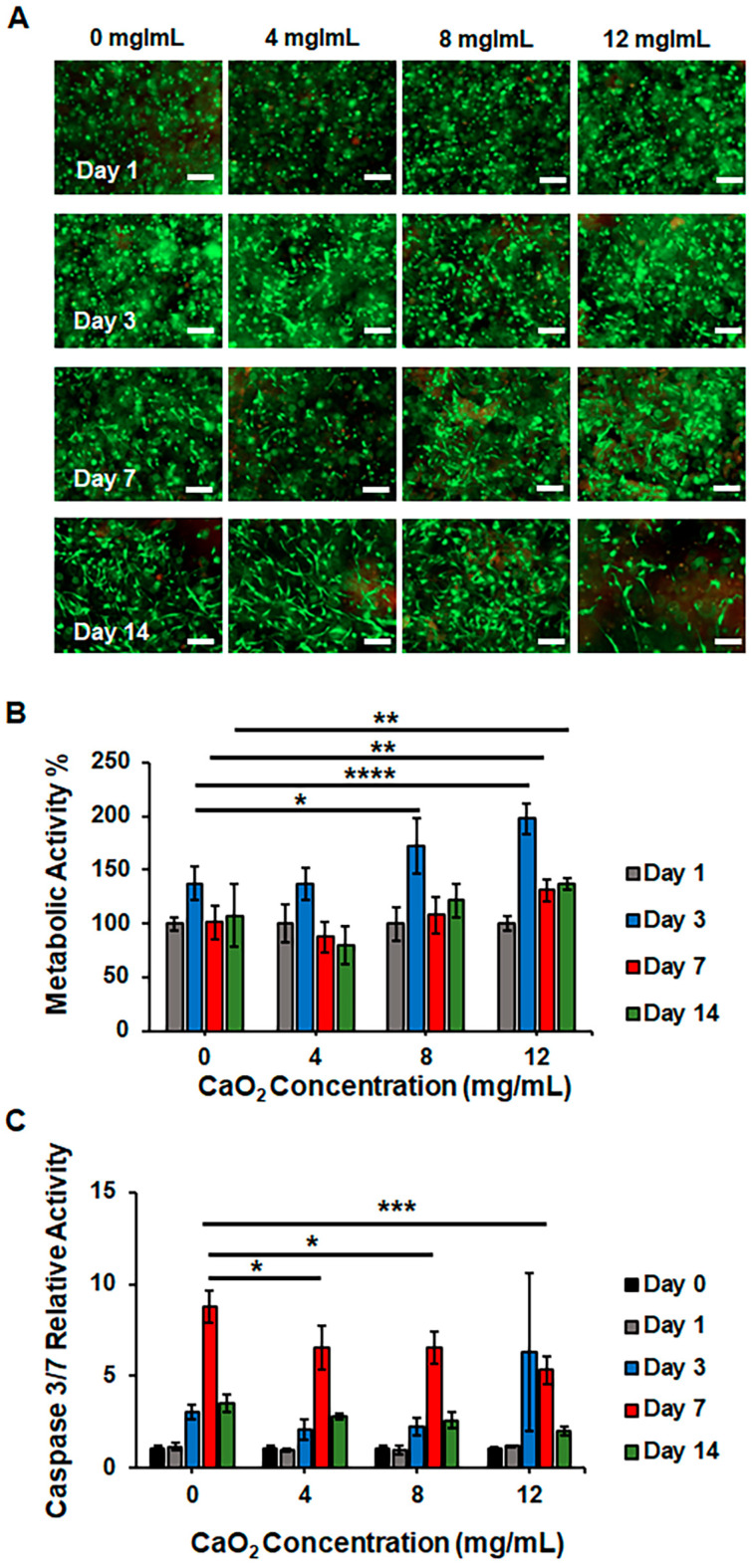
In vitro cytocompatibility analysis for oxygen-releasing 10% GelMA + 1% HAMA composite hydrogels encapsulating concentrations of CaO_2_ at 0, 4, 8, and 12 mg/mL. HDF cells were encapsulated in 5% (*w*/*v*) GelMA on a 96-well plate and co-cultured with oxygen-releasing hydrogels under hypoxia for 14 days. The cell culture media were supplemented with 1 mg/mL catalase: (**A**) Fluorescence microscopy images of HDFs showing live/dead (green/red) cells on days 1, 3, 7, and 14. (**B**) Metabolic activity and (**C**) Caspase 3/7 activity of HDFs were assessed over 14 days. Significant change between the groups was analyzed using two-way ANOVA which is shown as *p* < 0.05 (*), *p* < 0.01 (**), *p* < 0.001 (***), and *p* < 0.0001 (****).

HDFs encapsulated in a 5% (*w*/*v*) GelMA were incubated with a self-oxygenating composite hydrogel in a 96-well plate under hypoxic conditions for 14 days. The viability and proliferation profiles of the HDFs under hypoxia were evaluated using Calcein AM/PI staining and Alamar blue assays, respectively (Figure 5). As shown in the fluorescence microscopy images in Figure 5A, the cells were viable and homogeneously distributed in the 5% (*w*/*v*) GelMA on day 1. On day 3, regardless of the CaO_2_ concentration, HDFs showed high viability but started to elongate and form spindle-shaped morphology only in the wells with hydrogels containing 4, 8, and 12 mg/mL of CaO_2_. The cells showed higher viability in all conditions on days 7 and 14. The Alamar blue assay in Figure 5B provides quantitative information for the metabolic activity of HDFs under hypoxia. First, the metabolic activity on day 1 was determined by measuring the reduced resazurin dye fluorescence intensity in each well, and the average values of each condition were set to 100%. The values of fluorescence intensity of the later time points were normalized according to the results of day 1. The metabolic activity on day 3 showed an increasing tendency for all groups. The values were calculated as 137% (±15), 137% (±14), 173% (±26), and 198% (±15) for the wells where the pristine hydrogel and 4, 8, and 12 mg/mL CaO_2_-containing 10% GelMA + 1% HAMA composite hydrogels were present, respectively. As can be seen, the values were similar when the HDFs were incubated with the pristine and 4 mg/mL CaO_2_-containing hydrogels. The metabolic activity of the cells in the wells where 8 and 12 mg/mL CaO_2_-containing hydrogels were used was remarkably higher, as expected, due to the sufficient level of dissolved oxygen in the medium. However, a sudden decrease in the relative metabolic activity values was observed on day 7 for all conditions. The values were 101% (±16), 89% (±15), 108% (±17), and 131% (±11) for the pristine hydrogel and 4, 8, and 12 mg/mL CaO_2_-containing 10% GelMA + 1% HAMA composite hydrogels, respectively. This decrease can be attributed to the significant depletion of oxygen level in the medium, resulting in the established hypoxia, as shown in Figure 4B. However, the metabolic activity of the cells in the wells was higher when 12 mg/mL CaO_2_-laden hydrogels were used. On day 14, a decreasing trend in the metabolic activity of cells was observed in the wells where pristine and 4 mg/mL CaO_2_-laden composite hydrogels were present. However, the cells in the wells containing 8 and 12 mg/mL CaO_2_-laden hydrogels maintained metabolic activity on day 14, at a similar rate obtained on day 7.

To elucidate the unexpected metabolic activity decrease, a Caspase 3/7 assay was performed to monitor the relative cell death under hypoxia, as shown in Figure 5C. Similarly, the luminescence value on day 0 was used to normalize the values obtained at later time points and calculate the relative Caspase 3/7 activity. The HDFs in all conditions showed a similar rate of Caspase 3/7 activity until day 3. A drastic change in cell death was observed on day 7. The relative Caspase 3/7 activity significantly increased to 8.8 (±0.9), 6.6 (±1.2), 6.6 (±0.9), and 5.3 (±0.7) for the pristine hydrogel and 4, 8, and 12 mg/mL CaO_2_-containing hydrogels, respectively. As can be seen, the rates were lower in the wells where CaO_2_-laden 10% GelMA + 1% HAMA composite hydrogels were present compared to the pristine condition. These results support the findings of decreased metabolic activity. The significant decrease in the metabolic activity of cells on day 7 was due to cell death under hypoxia. However, the oxygen supplied through the decomposition of CaO_2_, especially at a concentration of at least 12 mg/mL, helped to maintain higher viability in HDFs compared to the pristine 10% GelMA + 1% HAMA composite hydrogels.

### 2.4. Antibacterial Activity of the Sprayable Composite Hydrogel

**Figure 6:**
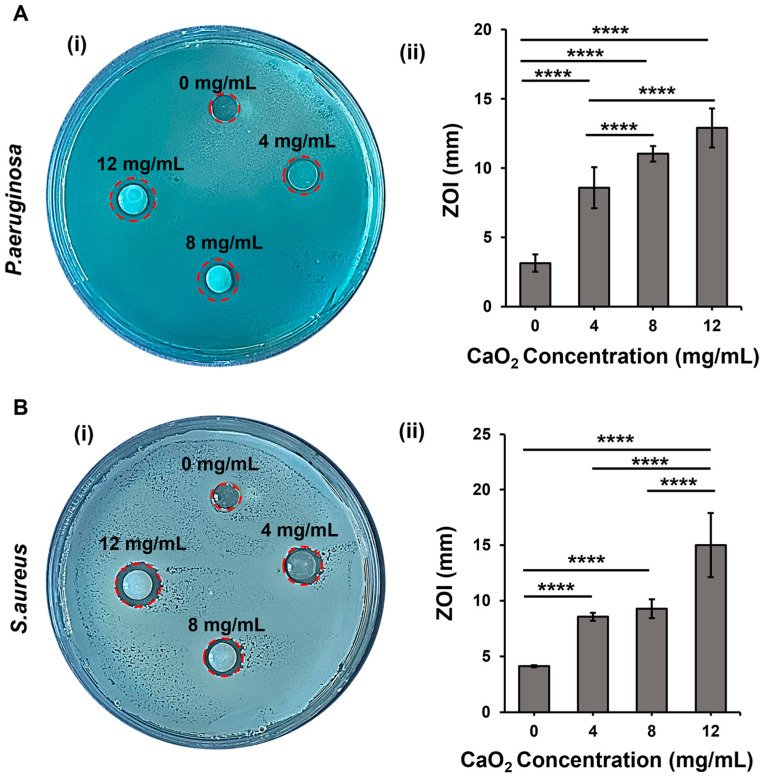
The antibacterial activity of the oxygen-releasing composite hydrogels was assessed using the ZOI test: (**A**) *P. aeruginosa* and (**B**) *S. aureus* were used as model microorganisms for skin infection. (i) The images show the ZOI formed around the composite hydrogels and (ii) quantitative analysis. Significant change between the groups was analyzed using one-way ANOVA which is shown as *p* < 0.0001 (****).

CaO_2_ exhibits antibacterial activity due to the release of H_2_O_2_ upon its decomposition in aqueous media [9]. We analyzed the antibacterial properties of our hydrogels using *P. aeruginosa* and *S. aureus*, which are most relevant to infections seen in skin wounds. We prepared the CaO_2_-laden hydrogels and evaluated their antibacterial properties using the zone of inhibition (ZOI) tests. Images in Figure 6A(i),B(i) clearly show the inhibition zones around the CaO_2_-containing hydrogels. The diameter of the ZOI on the *P. aeruginosa* plate was measured to be around 10.5, 11.8, and 12.8 mm for 4, 8, and 12 mg/mL CaO_2_-laden hydrogels, respectively (Figure 6A(ii)). The diameter size of the ZOI significantly increased with the increase in the concentration of CaO_2_, as expected. For *S. aureus*, 4 and 8 mg/mL CaO_2_ showed similar effects, and the size of the ZOI was measured as 10.5 mm. The ZOI was measured as 14 mm for the 12 mg/mL CaO_2_ concentration, which was significantly higher (Figure 6B(ii)) than the rest of the experimental conditions. These results demonstrate that our composite hydrogels display antibacterial action, and the effective activity changes depending on the CaO_2_ concentration.

## 3. Conclusions

In this study, we developed and characterized a sprayable wound dressing that exhibits self-oxygenating and antibacterial properties. The dressing is composed of a blend of methacrylate-modified gelatin and hyaluronic acid, which creates an optimal environment for skin regeneration. By using methylated precursors, we achieve instant chemical crosslinking and complete wound coverage when applying the hydrogel. Furthermore, the crosslinked hydrogel offers a stable microenvironment for extended healing and regeneration. The optimum sprayability of the composition in physiological conditions was obtained using a precursor solution consisting of 10% (*w*/*v*) GelMA and 1% (*w*/*v*) HAMA. This composite precursor solution had a flow property that allowed for easy and convenient layer-by-layer administration of the sprayable composite hydrogels. This aspect was demonstrated in ex vivo pig skin tissue. By adding different concentrations of CaO_2_ to the sprayable hydrogel formulation, we were able to achieve oxygen release for up to two weeks under hypoxic conditions. In an in vitro biomimetic model, human dermal fibroblast cells encapsulated in GelMA hydrogels showed that the stress induced by hypoxia was relieved by the oxygen release, as indicated by an increase in the metabolic activity rate and a decrease in the cell death rate. This significant effect was observed when the sprayable hydrogel formulation contained a minimum of 8 mg/mL of CaO_2_. The ZOI test demonstrated a concentration-dependent antibacterial activity of CaO_2_-laden sprayable hydrogels against common infectious bacteria found in skin wounds. These results suggest that the developed hydrogel-based wound dressing can potentially protect against bacteria during wound healing. Overall, we successfully developed a multifunctional sprayable hydrogel that is self-oxygenating and antibacterial. We believe that the composition of this sprayable hydrogel can be further enhanced by adding other biomolecules, possibly improving its functionality in wound healing and skin tissue engineering applications.

## 4. Materials and Methods

### 4.1. Materials

Porcine skin gelatin type A (300 Bloom) and catalase from bovine liver were purchased from Sigma-Aldrich (St. Louis, MO, USA). Sodium hyaluronate, research-grade with a range of Mw from 41 kDa to 60 kDa, was commercially obtained from Lifecore Biomedical (Chaska, MN, USA). Methacrylic anhydride (MA) was purchased from Polysciences (Warrington, PA, USA). Calcium peroxide (CaO_2_) and sodium sulfite were obtained from Thermo Scientific (Ward Hill, MA, USA). Dialysis membrane (12 to 14 kDa MWCO) was purchased from Spectrum™ (Stamford, CT, USA). Hydroxy-1-[4-(hydroxyethoxy) phenyl]-2-methyl-1-propanone (Irgacure 2959) was purchased from BASF Corporation (Florham Park, NJ, USA). Calcein AM/PI and Alamar blue assays were obtained from Invitrogen (Grand Island, NY, USA). The Caspase Glo 3/7 assay kit was obtained from Promega (Madison, WI, USA). Bacto^TM^ Tryptic Soy Broth (TSB) and Difco^TM^ Tryptic Soy Agar (TSA) were purchased from BD (Sparks, MD, USA).

### 4.2. Preparation of the Composite Sprayable Hydrogels

#### 4.2.1. Synthesis of Polymer Precursors

Porcine skin gelatin was used to synthesize gelatin methacrylate (GelMA) following the instructions in a previously published protocol [36]. Briefly, 10 g of gelatin was dissolved in 100 mL of Dulbecco’s phosphate-buffered saline (DPBS) while constantly stirring at 50 °C. Then, 8 mL of MA was added dropwise, and the mixture was continuously stirred for 4 h at 50 °C. Next, 300 mL of DPBS was added to the mixture to stop the methacrylation reaction. The resulting mixture was then dialyzed using a 12–14 kDa cutoff dialysis membrane. It was submerged in distilled water for a week while being stirred magnetically at 180 rpm and 40 °C. After dialysis, the solution was frozen overnight at −80 °C and freeze-dried for a week. The lyophilized samples were stored at −20 °C until further use.

Hyaluronic acid methacrylate (HAMA) was synthesized by following the previously described steps [25]. In brief, 1 g of sodium hyaluronate was dissolved in 100 mL of distilled water. Then, 1 mL of MA was added to this solution, and the reaction was carried out for 24 h at 4 °C. The pH of the solution was adjusted to between 8 and 10 using 5 M sodium hydroxide. The resulting reaction solution was then dialyzed against distilled water at 4 °C for three days. After dialysis, the solution was frozen at −80 °C and freeze-dried. The solidified samples were stored at −80 °C until further use.

#### 4.2.2. Development of the Sprayable Hydrogel

Various concentrations of prepolymer solutions, including 5%, 10%, and 15% (*w*/*v*) of GelMA, were prepared by dissolving GelMA in a 0.5% (*w*/*v*) Irgacure 2959 photoinitiator solution at 37 °C. We assessed the effectiveness, practicality, and ease of application of the hydrogel precursor solution to the wound area by testing different sprayer nozzles that dispensed small, medium, and high volumes of solutions.

First, the volumes of single spray from different sprayer nozzles were analyzed by utilizing various concentrations of the GelMA solution. Pressure was manually applied to the different nozzles attached to the bottles, with large (160 mL), medium (55 mL), and small (5 mL) volumes five times, and the streamed precursor solutions were collected in falcon tubes. The falcon tubes were then equilibrated to 37 °C, and centrifugation at 4000 rpm for 3 min was carried out to collect the sprayed precursor solution at the bottom of the tube. The volume of the precursor GelMA solutions was measured using a micropipette. The total volume was divided by 5 to measure the volume of a single spray. Three replicates were used for each sprayer nozzle, and the mean value was used for representation.

Secondly, we analyzed the area covered by a single spray of the precursor solution. The precursor solutions were filled into bottles equipped with sprayer nozzles that dispensed small volumes. To track the sprayed areas, we added green food dye to the precursor solutions. We manually applied pressure to the nozzles by holding the bottles at a specific angle and distance above a clean surface. The sprayed area was then measured using NIH ImageJ software v1.53k (Bethesda, MD, USA). We measured the area of the material sprayed onto the surface, which had a diameter of 34 mm, representing the largest diameter of the sprayed area. We conducted three replicates and used the average value for quantitative analysis.

Lastly, commercially available pig skin was used to analyze the spraying characteristics of various GelMA precursor solutions on ex vivo skin tissue. Different concentrations of GelMA were sprayed onto a 10 × 10 mm piece of skin tissue four times, totaling 300–375 µL. The crosslinking process involved the exposure of the tissue to UV light in the range of 320–500 nm for 20 s using an Omnicure S2000 at an intensity of 4 mW cm^−2^ (Excelitas Technologies Corp., Mississauga, ON, Canada). The layer-by-layer application of the precursor solutions was analyzed by spraying four times and applying UV light in each layer. After the UV-light exposure, images of the hydrogel constructs on pig skin were taken to determine the thickness of the hydrogel wound dressing.

### 4.3. Characterization of the Sprayable Composite Hydrogels

The composite hydrogels were characterized based on their mechanical and swelling properties. The prepolymer solution, which consisted of a mixture of 10% (*w*/*v*) GelMA and 1% (*w*/*v*) HAMA, was prepared in a 0.5% (*v*/*w*) PI solution at 37 °C. Briefly, 100 µL of the prepolymer solution containing 0, 4, 8, and 12 mg/mL of CaO_2_ was pipetted between spacers with a thickness of one mm. The solution was then crosslinked under UV light for 24, 50, 75, and 90 s, respectively, using an Omnicure S2000 at an intensity of 4 mW cm^−2^. Afterward, the prepared hydrogels were submerged in DPBS for 24 h to reach swelling equilibrium before analysis.

#### 4.3.1. Mechanical Properties

The compressive moduli of the sprayable composite hydrogels were measured using a Shimadzu EZ-L-XL universal testing machine (Columbia, MD, USA). Samples with a diameter of 8 mm and a thickness of 1 mm were prepared by punching. These samples were then placed on the plate and brought into contact with the upper compression plate. Stress–strain curves were plotted by conducting a compression test using a 10 N load cell at a speed of 0.5 mm/min. The elastic moduli of the hydrogels were calculated from the slope of the linear region between 5% and 20% of strain. Four replicates were tested for each condition.

#### 4.3.2. Swelling

The wet weight of the swollen hydrogels was recorded by placing them in preweighed Eppendorf tubes. Next, the hydrogels were frozen and freeze-dried for 24 h. The dried weight was then recorded, and the swelling ratio of the hydrogels was calculated by dividing their swollen weights by their dry weights. Four replicates were evaluated for each condition.

### 4.4. Cell Culture

Primary human dermal fibroblasts (HDFs) were used to assess the cytocompatibility and functionality of the oxygen-releasing sprayable hydrogels. HDFs (ATCC) were grown in the fibroblast basal medium (ATCC, Manassas, VA, USA) supplemented with fibroblast growth kit—low serum (ATCC), penicillin–streptomycin–amphotericin B solution (ATCC), and phenol red (ATCC), as recommended by the manufacturer. HDF cells were incubated in a humidified incubator at 37 °C with 5% CO_2_. Fresh complete media were added every 2–3 days.

### 4.5. Development of an In Vitro Model for Testing the Cytocompatibility and Functionality of Sprayable Hydrogels

An in vitro biomimetic model was developed to evaluate the oxygen-releasing performance of the composite hydrogels. First, HDFs attached to culture flasks were detached using trypsin treatment. Trypsin was then removed through centrifugation, and the number of cells was counted. Five million cells were precipitated through centrifugation and suspended homogeneously per mL of the 5% (*w*/*v*) GelMA precursor solution. Briefly, 40 µL of the suspension was pipetted into each well of a 96-well plate and crosslinked using UV light for 20 s at an intensity of 4 mW cm^−2^ using an Omnicure S2000. After crosslinking, 200 µL of fibroblast growth medium supplemented with 1 mg/mL of catalase was added to the hydrogels in the wells. The 96-well plates were placed in a humidified incubator at 37 °C and supplemented with 5% CO_2_ until the oxygen-releasing hydrogels were prepared.

In the next step, the composite hydrogels were prepared. Briefly, the precursor solutions, which included a mixture of 10% GelMA and 1% HAMA (*w*/*v*), were dissolved in a 0.5% (*v*/*w*) PI solution at 37 °C. Additionally, 4, 8, and 12 mg of CaO_2_ were suspended per mL of precursor solution. A 100 µL prepolymer solution was then pipetted between 1 mm thick spacers and crosslinked under UV light for 24, 50, 75, and 90 s, respectively. Hydrogels were prepared using a 6 mm diameter puncher and then placed into the wells on top of the 3D-encapsulated HDF cells in the 96-well plate. The plates were placed in a hypoxia chamber (StemCell Technologies, Vancouver, BA, Canada) and incubated at 37 °C.

#### 4.5.1. Dissolved O_2_ Measurement

A NeoFox oxygen-sensing probe (Ocean Optics Inc., Largo, FL, USA) was used to measure the dissolved oxygen within the media in a 96-well plate daily throughout a 12-day incubation period. The sensor was calibrated between 0% and 20.9% O_2_ before starting the daily measurements.

#### 4.5.2. Calcein AM/PI Staining

Live and dead cells were analyzed using Calcein AM/PI (green/red) staining. After incubating the cells in hypoxic conditions for 1, 3, 7, and 14 days, the GelMA hydrogel encapsulating HDFs was transferred from the 96-well plate to a glass-bottom plate. The cells were then stained with calcein AM/Pl according to the manufacturer’s instructions and imaged using fluorescence microscopy (ZEISS Axiocam 506, Jena, Germany).

#### 4.5.3. Alamar Blue and Caspase 3/7 Assays

The Alamar blue assay was used to assess the metabolic activity and relative viability of the HDF under hypoxic conditions on days 1, 3, 7, and 14. According to the manufacturer’s recommendation, the reaction mixture was prepared by mixing the fibroblast growth medium supplemented with 1 mg/mL of catalase with the Alamar blue reagent in a 1:10 ratio. The medium used was discarded from the wells but saved for use in the Caspase Glo 3/7 assay. Then, 200 µL of the prepared reaction mixture was added to the wells of a 96-well plate. The plate was placed in a hypoxia chamber and incubated for 4 h at 37 °C. The fluorometric changes in the media were measured at 560 nm/590 nm (Ex/Em) using the SPECTRA max Gemini XPS plate reader (San Jose, CA, USA). The values recorded on day 1 were used for normalization. Four replicates were used for each condition.

The apoptotic cell death under hypoxia was analyzed using the Caspase Glo 3/7 assay. The reaction substrate was mixed with the collected medium on days 1, 3, 7, and 14 in a 1:1 ratio. The mixture was then incubated for 45 min at room temperature shielded from light, as recommended by the manufacturer. A stop solution was added, and the luminescence of the solutions was recorded using the SPECTRA max Gemini XPS plate reader.

### 4.6. Antibacterial Activity

The antibacterial activity of the self-oxygenating composite hydrogels was determined by analyzing the zone of inhibition (ZOI). Gram-negative *P. aeruginosa* and Gram-positive *S. aureus* were used for the assessment. Briefly, microorganism cultures grown overnight in TSB were diluted in a 1:50 ratio. Then, 100 μL of the microorganism suspension was spread on TSA agar in Petri dishes. Hydrogels with an 8 mm diameter and 1 mm thickness cylindrical shape were placed on the agar Petri dishes and incubated at 37 °C overnight. The antibacterial activity was determined by measuring the ZOI around the hydrogel.

### 4.7. Statistical Analysis

The statistical significances were calculated using GraphPad Prism 6.0 (La Jolla, CA, USA). One- and two-way ANOVA analyses along with the Tukey test were performed, and *p* < 0.05 was considered statistically significant. Data were expressed as averages ± standard deviation (* *p* < 0.05, ** *p* < 0.01, *** *p* < 0.001, and **** *p* < 0.0001).

## Data Availability

All data and materials are available on request from the corresponding author. The data are not publicly available due to ongoing researches using a part of the data.

## Supplementary Materials

The following supporting information can be downloaded at: https://www.mdpi.com/article/10.3390/gels10030176/s1, Figure S1: 1H NMR spectra of GelMA, and HAMA; Figure S2: Contact angle measurement for the various concentrations of GelMA precursor solutions dropped on ex vivo pig skin; Figure S3: Comparison of the spraying characteristics between 10% GelMA and a mixture of 10% GelMA and 1% HAMA hydrogel precursor solutions is presented.
