# Supplementary material for: Development of a Sprayable Hydrogel-Based Wound Dressing: An In Vitro Model
Source: Gels. 2024 Mar 1;10(3):176. doi: 10.3390/gels10030176 (PMC10970460; doi:10.3390/gels10030176)
Supplement: Supplementary file 1 [file gels-10-00176-s001.zip › gels-2867074-supplementary.pdf]

*Article-Supplementary Material*

# Development of a Sprayable Hydrogel-Based Wound Dressing: An In Vitro Model

**Mine Altunbek [1], Mert Gezek [1,2], Maria Eduarda Torres Gouveia [1] and Gulden Camci-Unal [1,3,*]**

[1] Department of Chemical Engineering, University of Massachusetts Lowell, 1 University Avenue, Lowell, MA 01854, USA; mine_altunbek@uml.edu (M.A.); mert_gezek@student.uml.edu (M.G.); mariaeduarda_gouveia@student.uml.edu (M.E.T.G.)

[2] Biomedical Engineering and Biotechnology Program, University of Massachusetts Lowell, 1 University Avenue, Lowell, MA 01854, USA

[3] Department of Surgery, University of Massachusetts Medical School, 55 Lake Avenue North, Worcester, MA 01605, USA

* Correspondence: gulden_camciunal@uml.edu; Tel.: +1-978-934-3143

## Results

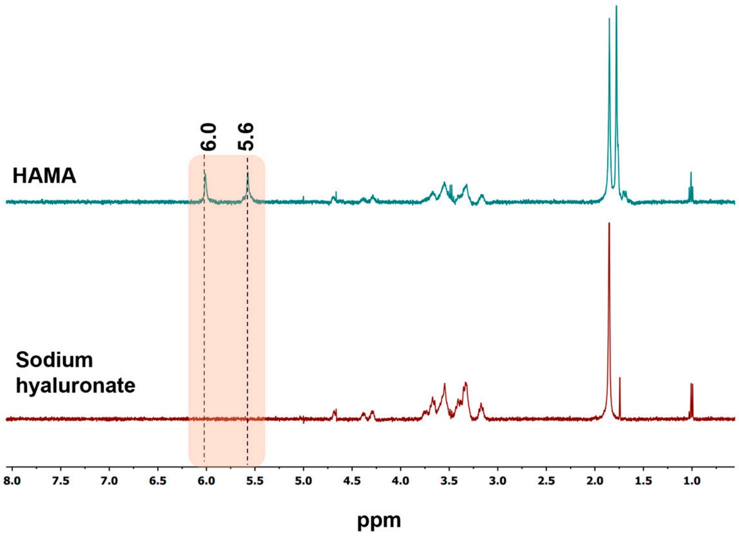


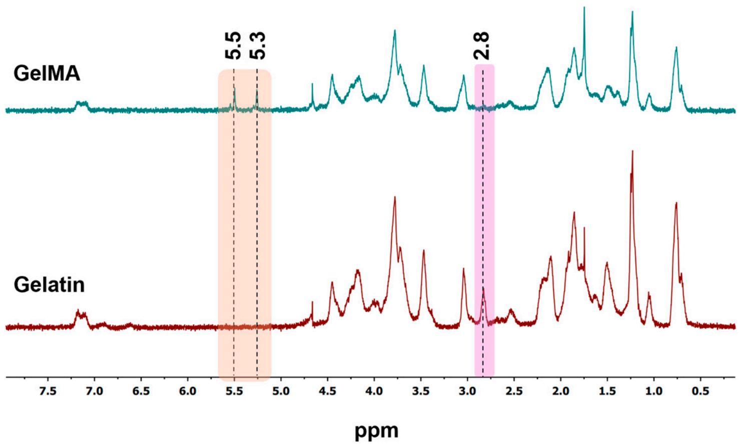


**Figure S1.** (A) $^1$H NMR spectra of sodium hyaluronate and HAMA. The peaks at ~5.6 and ~6.0 ppm show methacryloyl peaks which confirms methacrylate grafting on sodium hyaluronate. (B) $^1$H NMR spectra of gelatin and GelMA. The methacryloyl peaks were observed at ~5.3 and ~5.5 ppm. The disappeared peak around ~2.8 ppm in the gelatin is corresponding to the free amino groups which indicates the attachment of methacryloyl group.

Different concentrations of GelMA precursor solutions were dropped onto the *ex vivo* pig skin model and contact angle measurements were performed to relatively measure the viscosity dependent interaction of the sprayed hydrogels with the skin surface. The LB-ADSA method was used with ImageJ software to calculate the contact angle. A direct correlation was found between increasing concentration of GelMA and contact angle. The relationship between GelMA concentration and the viscosity of the material could be related to the contact angle on the *ex vivo* pig skin (Figure S2A). A 5 % (w/v) GelMA spread quickly on pig skin, and the contact angle was measured as 21°. The spread rates of 10 % and 15 % (w/v) GelMA were relatively low, and the contact angles were measured as 49° and 60°. These results indicate that viscosity is particularly important, as the less viscous hydrogel precursor can lead to quick spreading and leakage before crosslinking, while it can be difficult to evenly spread the high viscosity precursor solutions on the wound area. Since the 5 % (w/v) GelMA spread quickly and the spreading of 15 % (w/v) GelMA was very low, we selected the 10 % GelMA condition, which could be more suitable for *ex vivo* application.

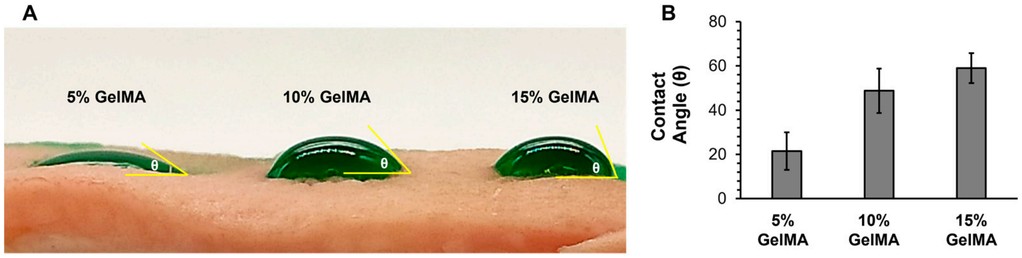


**Figure S2.** Contact angle measurement for the 5 %, 10 %, and 15% (w/v) of GelMA precursor solutions dropped on *ex vivo* pig skin. (A) Images of the droplets on the *ex vivo* pig skin and (B) quantitative analysis for the contact angles.

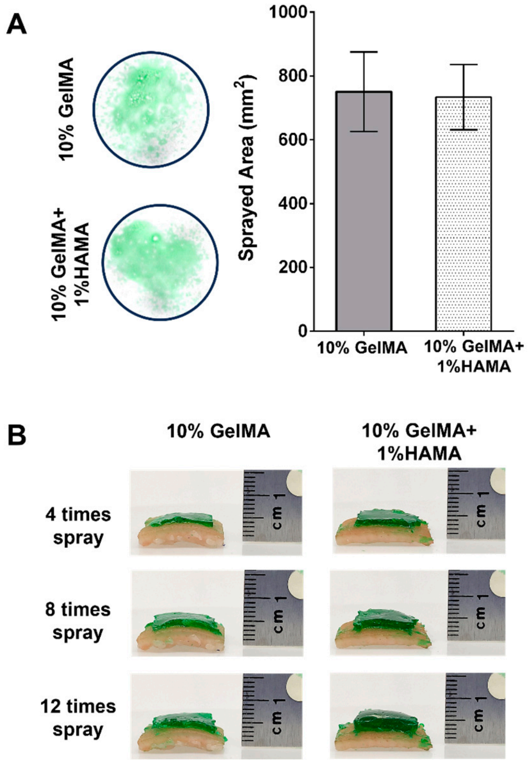


**Figure S3.** Comparison of the spraying characteristics between 10 % GelMA and a mixture of 10 % GelMA and 1 % HAMA hydrogel precursor solutions is presented. (A) Images of a single spraying area and quantitative analysis for the sprayed area are shown (B) Spraying of 10 % GelMA and the mixture of 10 % GelMA and 1 % HAMA hydrogel precursor solutions on pig skin using a layer-by-layer approach is demonstrated. Side views of the sprayed hydrogels with different numbers of sprays indicate that the thickness of the hydrogel dressing can be easily adjusted.
